# *The Plant Cell* welcomes 2024 Assistant Features Editors

**DOI:** 10.1093/plcell/koad263

**Published:** 2023-10-13

**Authors:** Nancy A Eckardt, Blake C Meyers

**Affiliations:** Senior Features Editor, The Plant Cell, American Society of Plant Biologists, USA; Editor-in-Chief, The Plant Cell, American Society of Plant Biologists, USA; Donald Danforth Plant Science Center, St. Louis, MO 63132, USA; Division of Plant Sciences and Technology, University of Missouri-Columbia, Columbia, MO 65211, USA

*The Plant Cell* is pleased to announce our Assistant Features Editors (AFEs) for 2024 (see Figure). *The Plant Cell* AFE program is now 6 years old! We started in July of 2017 with a cohort of 15 and have expanded to a group of 24, with about half rotating off and being replaced each year. The program provides AFEs with experience in writing for a broad audience, training in the peer review process, and networking opportunities with our editorial board, authors, and other AFEs. The AFEs, in turn, deliver valuable service to the journal, our authors, and the scientific community through their contribution of In Brief article highlights. The AFEs gain experience and coaching to improve their writing, receive editor and peer-review training, participate in editorial board meetings, and engage in other journal-related activities. In June of 2024, the AFEs will be supported to attend Plant Biology 2024 and the ASPB Centennial Celebration in Hawaii.

**Figure.**
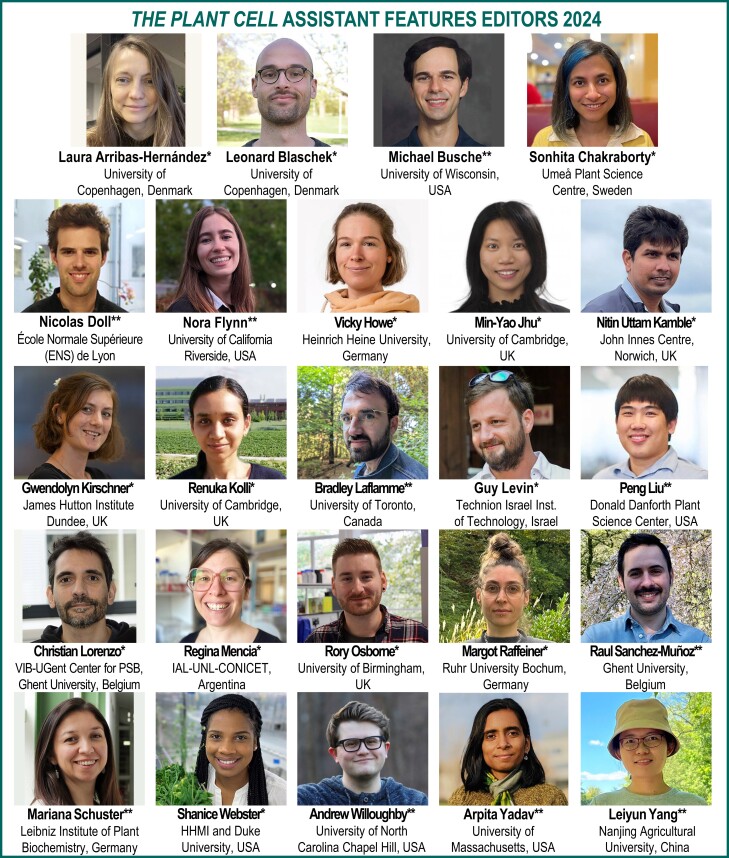
*The Plant Cell* Assistant Features Editors for 2024. *Joining January 2024. **Continuing.

We place a strong emphasis on the writing samples and other application materials to provide evidence that an applicant shows a commitment to science communication, an aptitude for storytelling and writing in an engaging manner, and the ability to easily grasp the major findings of work published in *The Plant Cell*. We also seek to bring on a diverse group who collectively can cover the range of topics published in the journal. Out of nearly 70 applications, it was extremely difficult to choose just 14 to join those continuing from last year. We thank all of these outstanding applicants for the time and effort put into their applications, and to those who wrote letters of recommendation. It gave us hope for the future to read of the accomplishments, dedication, and passion for plant biology of these early career researchers, even despite the career interruptions due to the COVID-19 pandemic. At the same time, we were sorry to turn away so many well-qualified applicants. After reading and rereading all 68 applications, we can safely say we would have enjoyed working with every one of these talented individuals. The future of plant science is in good hands!

In addition to our crew of 24 AFEs for 2024, we have nearly 60 alumni (including those stepping down at the end of 2023). Of these, the majority are group leaders heading their own labs or working in industry, government, or non-governmental organizations, many continue to conduct research as postdoctoral fellows or research associates, and several are science writers and editors. We provide a pathway to editorial board service for AFEs by following the careers of our alumni and each year encouraging the Editor-in-Chief to consider if there are 1 or 2 who might be invited to serve as Guest Editors in particular topical areas of need. Tom DeFalco (AFE January–December 2022; Assistant Professor of Biology at Western University, London, Ontario) has been a Guest Editor since the fall of 2022, and Sonali Roy (AFE July 2017–December 2019; Assistant Professor of Agricultural and Environmental Sciences at Tennessee State University, Nashville) was appointed Guest Editor in September 2023.

We offer our sincere thanks to the AFEs stepping down in 2024 for their service to the journal:

Mariana A.S. Artur, Postdoctoral Researcher, Laboratory of Plant Physiology, Wageningen University, Netherlands.

Carlisle Bascom, Jr., Postdoctoral Research Associate, University of New Hampshire, USA.

Marco Bürger, Research Associate, Salk Institute, USA.

Ching Chan, Assistant Professor, National Taiwan Normal University, Taiwan.

Lucas Frungillo, Lecturer in Plant Biotechnology, University of Edinburgh, UK.

Vera Gorelova, Postdoctoral Researcher, Wageningen University, Netherlands.

Sophie Hendrix, Tenure Track Assistant Professor, Hasselt University, Belgium.

Humberto Herrera-Ubaldo, Research Associate, University of Cambridge, UK.

Sara Lopez Gomollon, Lecturer in Plant RNA Biology, University of Kent, UK

Louis-Valentin Méteignier, Senior Researcher, INRAE, France.

Kutubuddin Ali Molla, Scientist, National Rice Research Institute, India.

Solène Moulin, Postdoctoral Research Fellow, Stanford University, USA.

Maryam Rahmati Ishka, Postdoctoral Researcher, Boyce Thompson Institute, USA.

Johan Zicola, Postdoctoral Researcher, Georg-August-University Göttingen, Germany.

It has been a pleasure working with all of you and getting to meet in person those who were able to make the trip to the editorial board meeting and Plant Biology 23 in Savannah, Georgia, in July 2023. We wish you all the best in your future endeavors.

*The Plant Cell* AFE program is highly competitive and a great addition to your C.V. Congratulations to all of our successful applicants! We look forward to working with this terrific group of early career researchers in 2024.

